# HCV-associated exosomes promote myeloid-derived suppressor cell expansion via inhibiting miR-124 to regulate T follicular cell differentiation and function

**DOI:** 10.1038/s41421-018-0052-z

**Published:** 2018-09-11

**Authors:** Lin Wang, Dechao Cao, Ling Wang, Juan Zhao, Lam Nhat Nguyen, Xindi Dang, Yingjie Ji, Xiao Y. Wu, Zheng D. Morrison, Qian Xie, Mohamed El Gazzar, Shunbin Ning, Jonathan P. Moorman, Zhi Q. Yao

**Affiliations:** 1 Center of Excellence in Inflammation, Infectious Disease and Immunity, James H. Quillen College of Medicine, East Tennessee State University, Johnson City, TN 37614 USA; 2 Division of Infectious, Inflammatory and Immunologic Diseases, Department of Internal Medicine, Quillen College of Medicine, ETSU, Johnson City, TN 37614 USA; 3 Center of Infectious Disease, Beijing Ditan Hospital, Capital Medical University, Beijing, 100015 China; 4 Center of Cadre Health Care, Beijing 302 Hospital, Beijing, 100000 China; 5 Hepatitis (HCV/HIV) Program, James H. Quillen VA Medical Center, Department of Veterans Affairs, Johnson City, TN 37614 USA

## Abstract

Virus-infected cells can regulate non-permissive bystander cells, but the precise mechanisms remain incompletely understood. Here we report that this process can be mediated by transfer of viral RNA-loaded exosomes shed from infected cells to myeloid-derived suppressor cells (MDSCs), which in turn regulate the differentiation and function of T cells during viral infection. Specifically, we demonstrated that patients with chronic hepatitis C virus (HCV) infection exhibited significant increases in T follicular regulatory (T_FR_) cells and decreases in T follicular helper (T_FH_) cells. These MDSC-mediated T-cell dysregulations resulted in an increased ratio of T_FR_/T_FH_ and IL-10 production in peripheral blood. Specifically, co-culture of MDSCs derived from HCV patients with healthy peripheral blood mononuclear cells (PBMCs) induced expansion of T_FR_, whereas depletion of MDSCs from PBMCs of HCV patients reduced the increases in T_FR_ frequency and IL-10 production, and promoted the differentiation of IFN-γ-producing T_FH_ cells. Importantly, we found that exosomes isolated from the plasma of HCV patients and supernatant of HCV-infected hepatocytes could drive monocytic myeloid cell differentiation into MDSCs. These exosomes were enriched in tetraspanins, such as CD63 and CD81, and contained HCV RNA, but exosomes isolated from patients with antiviral treatment contained no HCV RNA and could not induce MDSC differentiation. Notably, these HCV RNA-containing exosomes (HCV-Exo) were sufficient to induce MDSCs. Furthermore, incubation of healthy myeloid cells with these HCV-Exo inhibited the expression of miR−124, whereas reconstitution of PBMCs with miR−124 abolished the effects of HCV−Exo on MDSC induction. Taken together, these results indicate that HCV-associated exosomes can transfer immunomodulatory viral RNA from infected cells to neighboring immune cells and trigger MDSC expansion, which subsequently promotes T_FR_ differentiation and inhibits T_FH_ function. This study reveals a previously unrecognized path that represents a novel mechanism of immune dysregulation during chronic viral infection.

## Introduction

Hepatitis C virus (HCV) is a blood-borne pathogen characterized by a high rate (>80%) of chronic hepatitis, which can progress to liver cirrhosis and hepatocellular carcinoma—a leading cause for liver transplantation^1,2^. Notably, HCV has evolved numerous strategies to evade host immunity and harness virus persistence;^1,2^ thus, it has become an excellent model to study the mechanisms of virus-mediated host immune dysfunction and virus chronicity in humans. While the use of direct-acting antiviral (DAA) agents can efficiently clear HCV in the majority of infected individuals, this therapeutic cocktail faces new issues such as viral mutation, relapse and reinfection following therapy^3,4^. According to CDC (Centers for Disease Control and Prevention) reports, the number of HCV-related deaths reached an all-time high, surpassing 60 other nationally reportable infectious conditions combined, making hepatitis C the number one reportable infectious disease that kills people in the United States^5^. Similar to the issues inherent to HCV, the failure of the host to successfully manage many chronic infectious diseases, and to effectively respond to vaccines in the setting of viral infection, stem from our incomplete understanding of the pathogen–host interactions that can dampen host immunity and permit viral persistence.

CD4 T cells are central regulators of pathogen-specific immunity and vaccine response. They provide help to cytotoxic CD8 T cells and regulate humoral immune responses through interaction with B cells, but they can also participate in immunopathology directly via secretion of pro- and/or anti-inflammatory cytokines^6^. This functional versatility is achieved through differentiation of CD4 T cells into different lineages, such as T helper 1 (T_H_1), T helper 2 (T_H_2), T helper 17 (T_H_17), T follicular helper (T_FH_) and T regulatory (Treg) cells, including T follicular regulatory (T_FR_) cells^6^. While it is believed that specific immunological context critically influences the fate of T-cell differentiation, the precise mechanisms that drive T-cell lineage decisions and their roles in virus clearance or persistence remain largely unknown.

Myeloid-derived suppressor cells (MDSCs) are a heterogeneous population of immature myeloid cells that are generated due to aberrant myelopoiesis under various pathological conditions, such as cancer, inflammatory and infectious diseases^7–9^. These cells have gained special attention recently due to their potential to suppress immune responses, in particular, to induce regulatory T cells and to suppress the functions of effector T cells^10,11^. While MDSCs may contribute to immune homeostasis after infection via limiting excessive inflammatory processes, their expansion may be at the expense of pathogen elimination, and thus lead to persistent infection^9^. We and others have recently reported that MDSC expansion can inhibit T-cell functions by promoting Treg induction in multiple disease models, including chronic HCV/HIV infection^12–26^. However, the mediators that cause the expansion of MDSCs in the setting of chronic viral infection remain unclear. In addition, the role of MDSCs in regulating the differentiation and function of T follicular cells, an important subset of CD4 T cells that are responsible for regulation of antigen-specific B cell (vaccine) responses in the setting of HCV infection, has not been defined.

Exosomes are cell-derived, membrane-enclosed extracellular microvesicles (30–100 nm)^27,28^. Although the physiological function of exosomes remains unclear, these cell-released microvesicles have been implicated in serving as natural carriers to transfer materials (such as antigens, cytokines, messenger RNA (mRNA) or microRNAs (miRNAs)) among cells without direct cell-to-cell contact^29–31^. Mounting evidence suggests that exosomes have specialized functions and play a key role in processes such as intercellular signaling and immune regulation^32,33^. Notably, the human tetraspanin CD81 is enriched in exosomes and functions as the receptor for HCV E2 glycoprotein^34,35^. Indeed, HCV genomic materials can be released from infected hepatocytes into peripheral blood in the form of circulating exosomes, and these molecules can exploit the fusogenic capabilities of the exosomes with other cells to transmit HCV infection and to trigger plasmacytoid dendritic cell (pDC)-mediated innate immunity, even in the presence of neutralizing antibodies^36–39^. Whether HCV-associated exosomes (HCV-Exo) can induce MDSC expansion and contribute to T-cell dysregulation and viral persistence remains unknown.

To further investigate the characteristics of exosomes and their roles in MDSC, T_FH_ and T_FR_ dysregulation in HCV pathogenesis, we characterized the nature of HCV-Exo and their capacity in inducing MDSC expansion and T-cell dysfunction. We provide evidence that exosomes isolated from HCV-infected patients, but not DAA-treated subjects, carry HCV RNA and can induce MDSC expansion via inhibiting miR−124, which in turn promotes T_FR_ differentiation and interleukin-10 (IL-10) production. Depletion of MDSCs from peripheral blood mononuclear cells (PBMCs) of HCV-infected patients could reduce this aberrant T_FR_ and IL-10 induction, and promote T_FH_ cells to produce interferon-γ (IFN-γ). This study illustrates a novel mechanism of immune dysregulation during chronic viral infection.

## Results

### Phenotypic and functional characteristics of T_FR_ and T_FH_ cells in chronic HCV infection

**Fig. 1:**
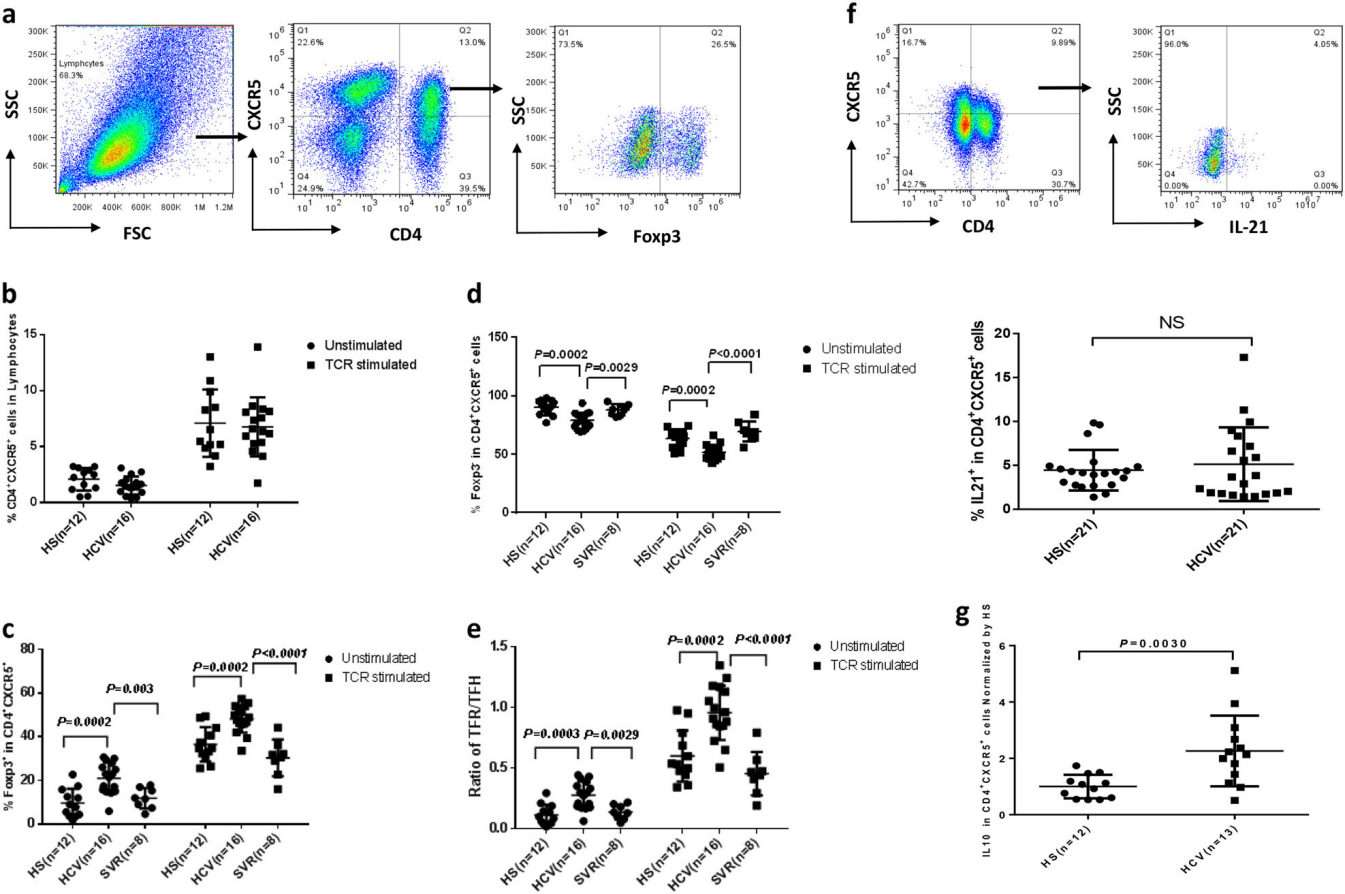
Phenotypic and functional characterization of T_FR_ and T_FH_ cells in HCV patients. **a** Representative dot plots analysis of T_FR_ and T_FH_ cells by flow cytometry. PBMCs were first gated on lymphocytes, and then T follicular cells (CD4^+^CXCR5^+^), followed by gating the Foxp3 marker to differentiate T_FR_ (CD4^+^CXCR5^+^Foxp3^+^) and T_FH_ (CD4^+^CXCR5^+^Foxp3^−^) cells, respectively. The values in the quadrants indicate the percentages of the related subsets. **b** Summary data for the percentage of T follicular cell (CD4^+^CXCR5^+^) frequencies in HS (*n* = 12) versus chronically HCV-infected patients (*n* = 16), in both unstimulated and TCR-stimulated conditions. **c** Summary data for the percentage of Foxp3^+^ cells in CD4^+^CXCR5^+^ cells in HS (*n* = 12) versus HCV-infected patients (*n* = 16) versus DAA-treated HCV subjects with SVR (*n* = 8) in freshly isolated and TCR-stimulated PBMCs. **d** Summary data for the percentage of Foxp3^−^ cells in CD4^+^CXCR5^+^ cells in HS versus HCV-infected patients versus DAA-treated subjects with SVR in freshly isolated and TCR-stimulated PBMCs. **e** The ratio of T_FR_/T_FH_ cells in HS versus HCV versus SVR in freshly isolated and TCR-stimulated PBMCs. **f** Representative dot plots and summary data for IL-21 expression in CD4^+^CXCR5^+^ T follicular cells. **g** Pooled data of IL-10 expression in CD4^+^CXCR5^+^ T follicular cells from HCV patients (*n* = 13) normalized by HS (*n* = 12). Each symbol represents an individual subject, and the horizontal bars represent median with interquartile. *P* value with significant difference is shown in each panel; NS no significance

CD4^+^CXCR5^+^ T follicular cells are an important subset of T cells involved in germinal center formation and regulation of the humoral immune responses^40^. While recent studies in humans have improved our understanding of T_FH_ cell biology^41–43^, the cell frequency, cytokine profile and their role in HCV infection remain controversial^44,45^. The discrepancy of these results is likely due to the different markers and methods used (e.g., using PD-1 or CXCR3 in addition to the CD4 and CXCR5) by different investigators to test HCV patients with different disease stages. Interestingly, recent studies have shown that CD4^+^CXCR5^+^ T follicular cells can be further classified into T_FR_ (CD4^+^CXCR5^+^Foxp3^+^) and T_FH_ (CD4^+^CXCR5^+^Foxp3^−^), depending on the level of forkhead box protein 3 (Foxp3) expression. To further define the role of these subsets of T cells in chronic HCV infection, we analyzed the cell frequency, cell ratio and cytokine secretion of T_FH_ and T_FR_ in the peripheral blood of HCV-infected individuals versus healthy subjects (HS). As shown in Fig. 1a (representative dot plots of the gating strategy) and Fig. 1b (summary data of the flow cytometry), the frequencies of CD4^+^CXCR5^+^ T follicular cells, under both unstimulated and anti-CD3/CD28-stimulated conditions, were slightly lower in PBMCs derived from chronically HCV-infected patients compared to age-matched HS, with no statistical difference. The analysis of Foxp3 expression in CD4^+^CXCR5^+^ subsets under both unstimulated and TCR-stimulated conditions revealed significant increases in T_FR_ cells (Fig. 1c), and concomitant decreases in T_FH_ cells (Fig. 1d) in HCV-infected patients compared to HS. Notably, HCV patients who received DAA treatment restored T_FR_ and T_FH_ cell frequencies after achieving sustained virological response (SVR). In addition, HCV-infected patients exhibited a significant increase in T_FR_/T_FH_ ratio in peripheral blood, which could be completely reversed by the DAA treatment (Fig. 1e).

To characterize the function of T follicular cells during HCV infection, we analyzed the intracellular cytokine expression in CD4^+^CXCR5^+^ T cells by flow cytometry following phorbol myristate acetate and kanamycin ex vivo stimulation for 5 h. Interestingly, while no significant difference in IL-21 production was observed (Fig. 1f), a significant increase in IL-10 production was detected in CD4^+^CXCR5^+^ T follicular cells derived from HCV patients versus HS (Fig. 1g). These results suggest that chronic HCV infection is associated with a decrease in T_FH_ cells with relatively low IL-21 expression on a per-cell basis and an increase in T_FR_ cells with remarkably elevated IL-10 production. However, no correlations were found among the T_FH_ and T_FR_ cell frequencies or their cytokine productions with HCV genotype and viral load in chronically HCV-infected patients (data not shown).

### MDSCs inhibit T_FH_ cell function and promote T_FR_ cells during chronic HCV infection

**Fig. 2:**
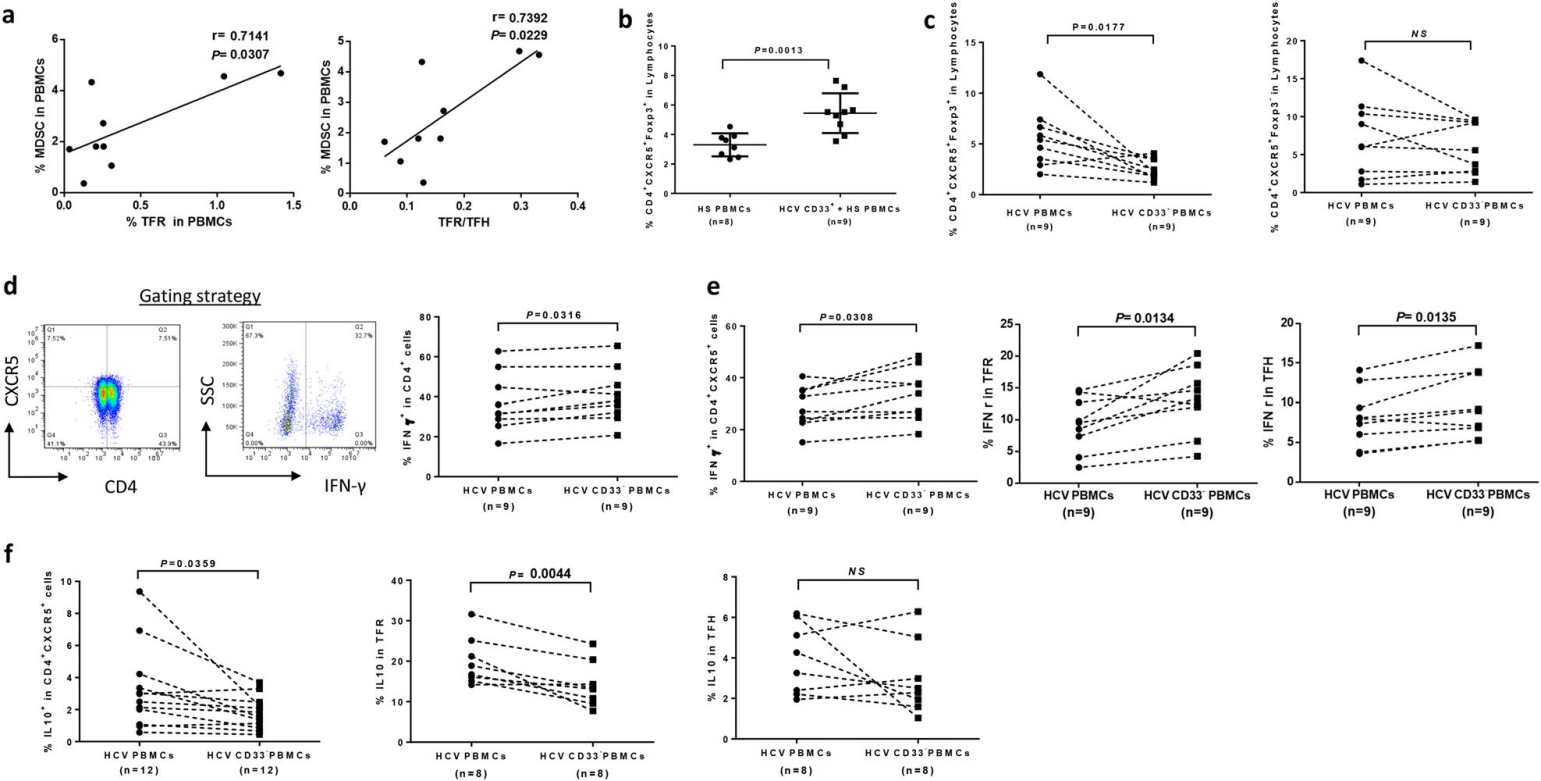
MDSCs from HCV patients promote Foxp3^+^ T_FR_ cell differentiation and suppress IFN-γ expression by Teff cells. **a** Correlation of MDSCs with T_FR_/T_FH_ cells in peripheral blood of HCV patients. The frequencies of MDSC (CD14^+^CD33^+^HLA-DR^−^), T_FR_ (CD4^+^CXCR5^+^Foxp3^+^) and T_FH_ (CD4^+^CXCR5^+^Foxp3^−^) cells in PBMCs of 9 HCV-infected patients were simultaneously examined by flow cytometry, and then analyzed by Pearson Correlation. *R* and *P* values are shown above the summary data. **b** CD33^+^ myeloid cells from HCV patients induce T_FR_ cell generation. Healthy PBMCs were co-cultured with or without CD33^+^ myeloid cells derived from HCV patients for 5 days, followed by flow cytometric analysis for the percentage of T_FR_ cell (CD4^+^CXCR5^+^Foxp3^+^) frequency in lymphocytes. **c** Reducing T_FR_ cells, but not T_FH_ cells, by depletion of CD33^+^ myeloid cells from PBMCs derived from HCV patients. HCV-derived PBMCs, with or without MDSC depletion, were cultured in vitro for 5 days, followed by flow analysis for CD4^+^CXCR5^+^Foxp3^+^ T_FR_ and CD4^+^CXCR5^+^Foxp3^−^ T_FH_ cells. **d** Representative dot plots and summary data of flow cytometric analysis of IFN-γ production by CD4^+^ cells in PBMCs with or without CD33^+^ myeloid cell depletion. **e** Depletion of CD33^+^ myeloid cells from PBMCs of HCV patients improves IFN-γ production by T follicular cells, T_FR_ and T_FH_ cells. HCV PBMCs, with or without CD33^+^ myeloid cell depletion, were cultured ex vivo in the presence of stimulation for 5 days, followed by immune staining and flow analysis for IFN-γ production by CD4^+^CXCR5^+^ T follicular cells as well as CD4^+^CXCR5^+^Foxp3^+^ T_FR_ and CD4^+^CXCR5^+^Foxp3^−^ T_FH_ cells. **f** Depletion of CD33^+^ myeloid cells from PBMCs of HCV patients reduces IL-10 production by T follicular cells and T_FR_ cells, but not by T_FH_ cells. PBMCs isolated from chronic HCV patients, with or without depletion of CD33^+^ myeloid cells, were cultured ex vivo in the presence of anti-CD3/CD28 stimulation, followed by flow cytometric analysis for IL-10 expression in CD4^+^CXCR5^+^ T cells as well as CD4^+^CXCR5^+^Foxp3^+^T_FR_ and CD4^+^CXCR5^+^Foxp3^−^ T_FH_ cells. *P* values are shown above each panel

We have recently shown an expansion of MDSCs during chronic HCV and HIV infections, which could inhibit T-cell function (IFN-γ production) via promoting Treg differentiation^20,25^. Since T_FR_ is a specialized subset of Treg, we hypothesized that the observed increases in T_FR_ might be due to the expansion of MDSCs in chronic viral infection. To test this hypothesis, we simultaneously examined the frequencies of MDSC, T_FR_ and T_FH_ cells in PBMCs from 9 HCV-infected patients and then analyzed their associations by Pearson's correlation. As shown in Fig. 2a, there was a clear positive correlation between MDSC and T_FR_ cell frequencies in PBMCs derived from HCV-infected patients. Additionally, the percentage of MDSCs positively correlated with the ratio of T_FR_/T_FH_ in PBMCs of HCV patients. However, there was no correlation between MDSC and T_FH_ cell frequencies in PBMCs of HCV patients (data not shown). To further clarify the relationship between MDSCs and T_FR_/T_FH_ cells, we purified CD33^+^ myeloid cells from HCV-infected patients and incubated them with healthy PBMCs for 3 days, followed by measuring the percentage of CD4^+^CXCR5^+^Foxp3^+^ T_FR_ frequencies within lymphocytes. As shown in Fig. 2b, co-culture of HCV-derived CD33^+^ myeloid cells with healthy PBMCs resulted in a significant increase in T_FR_ induction compared with healthy PBMCs cultured without HCV MDSCs. In contrast, depletion of CD33^+^ myeloid cells from HCV PBMCs resulted in a significant decrease in T_FR_ cells, but not T_FH_ cells, compared with the HCV PBMCs cultured with MDSCs (Fig. 2c). These results are in line with our finding that there is a clear correlation between MDSC and T_FR_ cells, but not T_FH_ cells, in the peripheral blood of HCV patients. As we had previously reported^20,25^, removal of MDSCs from HCV PBMCs enhanced overall CD4 T effector cell function, as evidenced by the significant increases in IFN-γ production in total CD4^+^ T cells (Fig. 2d). Furthermore, depletion of MDSCs from HCV PBMCs led to a significant increase in IFN-γ secretion in CD4^+^CXCR5^+^ T follicular cells, as well as in T_FR_ and T_FH_ cells (Fig. 2e). Moreover, depletion of MDSCs from HCV PBMCs led to a significant reduction in IL-10 expression in CD4^+^CXCR5^+^ T follicular cells and in T_FR_ cells, but not in T_FH_ cells (Fig. 2f). Taken together, these results suggest that HCV-associated MDSCs can increase T_FR_ cell number and inhibit the function of T_FH_ cells during chronic viral infection.

### HCV-Exo induce MDSC expansion

**Fig. 3:**
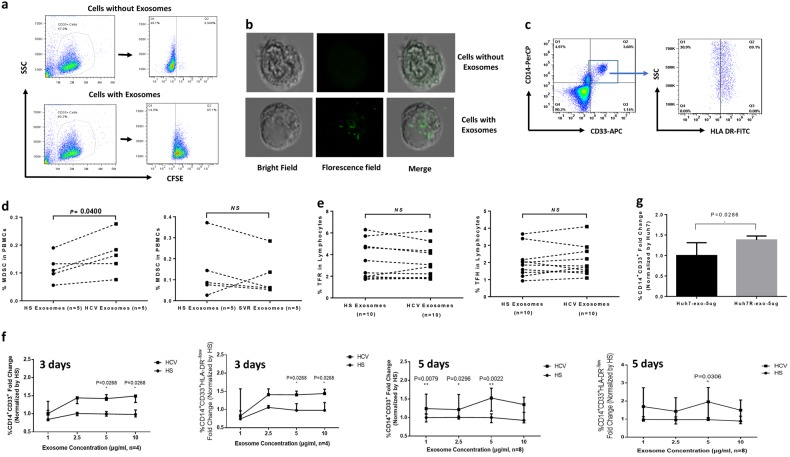
Exosomes are taken up by myeloid cells and promote MDSC development. **a** Representative dot plots of flow cytometric analysis of the uptake of CFSE-labeled exosomes by the purified myeloid cells. Isolated exosomes were labeled with CFSE, incubated with the purified CD33^+^ myeloid cells, followed by flow cytometric analysis for the uptake of CFSE-labeled exosomes. Cells incubated without exosomes served as control. **b** Confocal microscopic analysis of the uptake of CFSE-labeled exosomes by CD33^+^ myeloid cells. Cells incubated without exosomes served as negative control. A typical cell with bright light field, florescence field and merge is shown. **c** Representative dot plots showing the gating strategy for flow cytometric analysis of CD14^+^CD33^+^HLA-DR^−^ MDSCs. **d** Summary data of flow cytometric analysis of the MDSC frequencies in PBMCs exposed to HS-Exo, HCV-Exo and SVR-Exo for 3 days. **e** Summary data of flow cytometric analysis of the T_FR_ and T_FH_ cell frequencies in PBMCs exposed to HS-Exo, HCV-Exo and SVR-Exo for 3 days. **f** Summary data of flow cytometric analysis of the kinetic changes in monocytic myeloid cells and MDSCs in PBMCs treated with HCV-Exo and HS-Exo in a time- and dose-dependent manner. Those with statistically significant fold changes of M-MCs and M-MDSCs induced by HCV-Exo (normalized by HS-Exo) are marked as *P < 0.05 or **P < 0.01. **g** Induction of monocytic myeloid cells by exosomes isolated from the supernatants of HCV-infected (Huh7R) and uninfected (Huh7) hepatocytes. The data were reproducible in three independent experiments. *P < 0.05, **P < 0.01

We and others have recently reported a significant expansion of monocytic MDSCs (M-MDSCs) in chronic HCV infection;^21–25^ however, the factors that cause MDSC expansion during HCV infection remain unclear. Exosomes are small membrane vesicles released into the extracellular milieu by many cell types; they have fusogenic capability to transfer materials among cells, and are capable of regulating immune responses without direct cell–cell contact^26–34^. Thus, we determined whether exosomes isolated from the plasma of HCV patients can induce the expansion of MDSCs. We first investigated whether the exosomes can gain access into the myeloid cells. To this end, exosomes were isolated from HCV plasma, labeled with carboxyfluorescein succinimidyl ester (CFSE) and incubated with healthy CD33^+^ myeloid cells for 1 h, followed by flow cytometric and confocal microscopy analyses. As shown in Fig. 3a, the majority of CD33^+^ cells incubated with the CFSE-labeled exosomes showed fluorescence signal. The presence of CFSE-labeled exosomes in the CD33^+^ myeloid cells was confirmed by confocal microscopy (Fig. 3b).

We then determined whether HCV-Exo have any biological effect on the blood cells. To this end, exosomes were isolated from the plasma of 5 HCV-infected patients, 5 age-matched HS and 5 DAA-treated patients with SVR, incubated with healthy PBMCs for 3 days and subjected to flow cytometry to measure MDSC expansion. As shown in Fig. 3c (the gating strategy for CD14^+^CD33^+^HLA-DR− MDSCs) and Fig. 3d (summary data of the percentage of MDSCs in PBMCs exposed to exosomes), HCV-Exo significantly induced M-MDSC differentiation compared to HS-Exo, whereas there was no difference in MDSC induction by exosomes isolated from HS and DAA-treated patients with SVR, suggesting a role for active HCV infection in exosome-mediated MDSC induction. We also analyzed the effect of exosomes on T_FR_ and T_FH_ differentiation with PBMCs under the same treatment. However, we did not observe any direct effect of HCV-Exo on T_FR_ and T_FH_ differentiation (Fig. 3e), suggesting that the observed T-cell dysregulation may be due to a secondary effect from exosomes on MDSCs, rather than on T cells within such a short period of exposure time as modeled in this system.

To further examine the kinetic changes of MDSC induction by HCV-Exo, we incubated PBMCs with exosomes at varying concentrations for different times, and then analyzed by flow cytometry for the frequencies of CD14^+^CD33^+^ monocytic myeloid cells (M-MCs) as well as CD14^+^CD33^+^HLA-DR^−^ M-MDSCs. As shown in Fig. 3f, after 3–5 days in culture, HCV-Exo increased the numbers of both M-MCs and M-MDSCs in a time- and dose-dependent manner compared to the controls treated with HS-Exo. Since chronically HCV-infected patients often have other medical conditions (such as diabetes mellitus, rheumatoid arthritis) which may affect the host immunity and confound the results, we also assessed the effect of exosomes isolated from the culture supernatant of HCV-infected versus non-infected Huh7 hepatocytes on healthy PBMCs. As shown in Fig. 3g, a significant expansion of CD14^+^CD33^+^ M-MCs was observed when PBMCs were incubated with exosomes derived from HCV^+^ Huh7R compared to HCV^−^ Huh7 cells. Together, these results suggest that HCV-Exo can be taken up by myeloid cells to induce MDSC expansion during viral infection.

### HCV-Exo contain HCV RNA

**Fig. 4:**
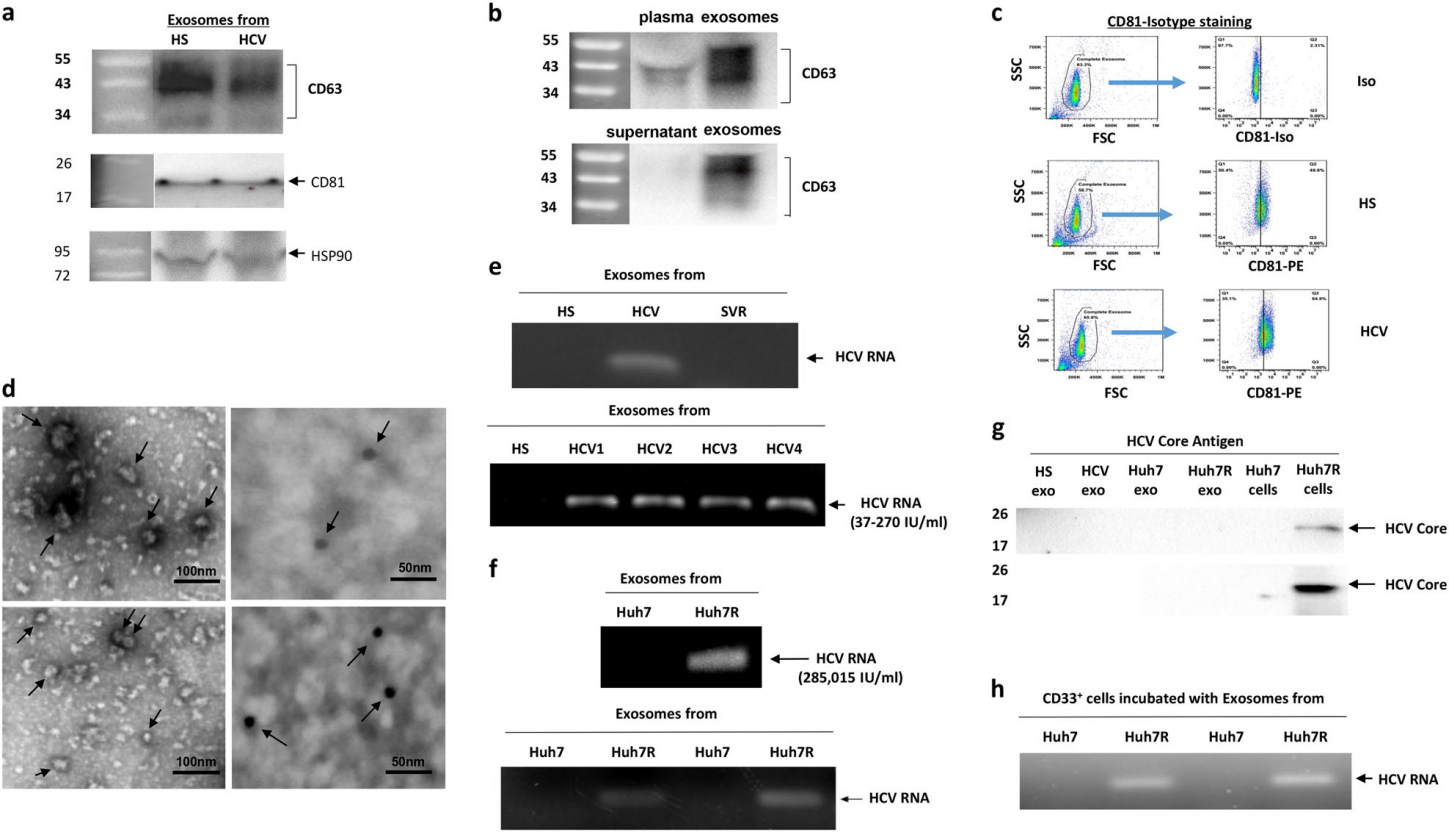
Characterization of exosomes isolated from HCV patient plasma and HCV-infected cells. **a** Identification of CD63, CD81 and HSP90 expressions in exosomes isolated from the plasma of HCV patients and HS (with exoEasy Maxi Kit) by immunoblotting. **b** Evaluation of CD63 concentration in the plasma and purified exosomes isolated from the same subject, or in the supernatant and enriched exosomes with exoEasy Maxi Kit. The same amount of proteins were loaded in the immunoblotting. **c** Detection of CD81 expression in exosomes (purified by CD81-coated beads) by flow cytometric analysis. **d** Electron microscope observation of exosomes. Exosomes isolated from the supernatant of HCV^+/−^ Huh7 cells by differential centrifugation were immuno-stained with or without anti-CD81 antibody, followed by secondary antibody labeled with 12 nm gold particles, then subjected to IEM observation. Arrowed structures represent exosomes without gold labeling (left panel) and exosomes associated with gold particles (right panel). Bars = 50 or 100 nm as indicated. **e** Detection of HCV RNA by RT-PCR in exosomes purified by sequential centrifugation from HCV^+/−^ subjects (upper panel) and quantification of their copy numbers in exosomes isolated from 1 ml plasma of HCV-infected patients by real-time RT-PCR. **f** Detection of HCV RNA by real-time RT-PCR in exosomes isolated by the centrifugation method from culture supernatants of HCV^+/−^ Huh7 cells. HCV RNA copy number, shown as IU/ml supernatant-derived exosomes, is shown in the upper panel. Repeated experiments by RT-PCR measuring HCV RNA in exosomes purified by CD81-coated beads from the culture supernatant of HCV-infected (Huh7R) and -uninfected (Huh7) hepatocytes are shown in the lower panel. **g** Detection of HCV Core protein in exosomes isolated from the plasma of HCV^+/−^ subjects or from the supernatants of HCV^+/−^ Huh7 cells by exoEasy Maxi Kit followed by CD81-coated beads purification methods. Lower panel show exosomes isolated from the supernatants of HCV^+/−^ Huh7 cells by the sequential centrifugation method. Cell lysate from Huh7 and Huh7R hepatocytes served as negative and positive controls. **h** Repeated experiments by RT-PCR measuring HCV RNA in CD33^+^ myeloid cells incubated with exosomes isolated from the supernatants of HCV^+/−^ Huh7 cells overnight

Since exosomes are enriched in CD81, the receptor for HCV E2 envelope protein, we hypothesized that HCV-infected hepatocytes may shed viral particles into the patient plasma in the form of exosomes to act as natural carriers to transfer HCV RNA from infected hepatocytes to blood cells. To test this possibility, we isolated exosomes from the plasma of HCV-infected patients and HS. We first characterized the nature of purified exosomes for the presence of their markers, CD63 and CD81, using immunoblotting. As shown in Fig. 4a, CD63 (a glycosylated protein of 30–60 kDa) and CD81 (22–26 kDa) bands were detected in HS-Exo as well as HCV-Exo. Since exosomes from HCV patients have been shown to transmit HCV infection via viral RNA in complex with heat shock protein 90 (HSP90) and miRNA^38^, we measured HSP90, which was found to be positively expressed in HS-Exo as well as HCV-Exo. We further measured multiple miRNAs, including miR−124, miR-125 and miR-29, which were shown to be significantly dysregulated in CD33^+^ myeloid cells during HCV infection^26^, but their levels did not differ in HS-Exo and HCV-Exo (data not shown). Notably, these plasma-derived exosomes, as well as those purified from the supernatants of cultured Huh7 hepatocytes, were significantly enriched in tetraspanins, as evidenced by stronger CD63 bands compared to the same amount of plasma or supernatant proteins loaded in the immunoblotting (Fig. 4b). In addition, we purified exosomes from the plasma of HCV and HS using a specific anti-CD81 magnetic beads, followed by flow cytometric analysis of the bead-coated exosomes using a phycoerythrin (PE)-labeled anti-human CD81 antibody. Again, these magnetic bead-purified exosomes from both HS and HCV plasma were enriched in CD81 tetraspanin (Fig. 4c). To visualize the structure and identify the exosomal marker CD81, we performed immuno-electron microscopy (IEM) on the purified exosomes, which were stained with or without mouse anti-huCD81 antibody, followed by anti-mouse IgG gold particles (12 nm in diameter). As shown in Fig. 4d, we observed exosome vesicles, ranging from 30 to 90 nm (left panel without gold labeling), with CD81-gold particles bound to their membrane surface (right panel with gold labeling).

Since HCV E2 envelope protein binds human tetraspanin CD81 with high affinity, and CD81 is enriched in exosomes released from cells including hepatocytes^34,35^, we next sought to determine whether exosomes isolated from the plasma of HCV-infected patients or culture supernatants of HCV-infected hepatocytes contain HCV RNA or proteins that may act as inducers of MDSC development. To this end, we isolated the exosomes using the sequential centrifugation method^36^ and performed reverse transcription-polymerase chain reaction (RT-PCR) for HCV RNA and western blot for HCV E1, E2 and Core proteins. As shown in Fig. 4e, HCV RNA was found in exosomes isolated from the plasma of HCV-infected patients, but not in those who received DAA treatment with SVR or from HS (upper panel). We also measured the viral load in purified exosomes by real-time RT-PCR and amplified 37–270 international unit (IU) per ml of plasma-derived exosomes from HCV patients (lower panel). As reported by other investigators^36^, this immunosorting method does not allow quantification of the exosomes purified out of the total plasma exosome pool, and because we do not know what fractions of plasma-derived exosomes are released by HCV-infected cells, it is difficult to estimate how much HCV RNA is associated with exosomes in patients’ plasma. In addition to exosomes derived from patient plasma, we isolated exosomes by ultracentrifugation from the supernatants of HCV^+/−^ Huh7 cells. As shown in Fig. 4f upper panel, HCV RNA was only detected in the exosomes isolated from the supernatants of HCV-infected Huh7R cells, with a copy number of 285,015 IU/ml supernatant-derived exosomes, but not in those from non-infected Huh7 cells. Because HCV virions and exosomes have very similar buoyant densities in sucrose gradient, there was a concern that the traditional ultracentrifugation method is insufficient for separating pure exosomes free of virus contamination. To overcome this limitation and to exclude other microparticles or free HCV contamination, we employed a Total Exosome RNA and Proteins Isolation Kit followed by a CD81-conjugated immune-magnetic bead isolation method to purify exosomes from culture supernatants of HCV-infected and -uninfected hepatocytes. As shown in Fig. 4f lower panel, HCV RNA was detected in exosomes purified from the supernatants of HCV-infected Huh7R cells, but not in exosomes isolated from non-infected Huh7 culture media. Similar to previous reports by other investigators who failed to detect viral proteins in these exosomes^36^, we also could not detect any HCV proteins in exosomes isolated from the plasma of HCV patients or the supernatant of HCV-infected Huh7 cells, either by CD81-bead purification (upper panel) or by sequential centrifugation isolation method (lower panel), although HCV core protein could be detected in the cell lysates from HCV^+^ Huh7 cells by western blotting (Fig. 4g). Also, we could not detect β-actin or glyceraldehyde 3-phosphate dehydrogenase (GAPDH) RNA or their proteins in exosomes by either isolation method. Since HCV RNA-containing exosomes can trigger pDC innate immunity^37^ and pDCs function as sentinels of viral infection, predominantly via recognition of single-stranded RNA by endosomal Toll-like receptors (TLRs)^46^, we thus hypothesized that HCV RNA might associate with TLRs in exosomes to be secreted by hepatocytes into the circulation. However, while we detected HCV RNA in the HCV-Exo, we could not detect TLR-3, -7 and -9 in the HCV-Exo, perhaps due to the low sensitivity of this detection method and/or very low levels of HCV-associated TLR proteins in these purified exosomes.

We next asked whether HCV-Exo could transfer viral RNA into myeloid cells to drive MDSC differentiation. As shown in Fig. 4h, real-time RT-PCR provided mechanistic evidence that CD33^+^ myeloid cells contain HCV RNA after incubation overnight with exosomes derived from HCV-infected Huh7R cells, but not in myeloid cells incubated with exosomes isolated from non-infected Huh7 cells. These results suggest that the fusogenic capabilities of exosomes could deliver HCV RNA into myeloid cells and trigger MDSC induction.

### HCV-Exo induce MDSC expansion through a miR-124-mediated signaling pathway

CD81, scavenging receptor class B member I (SR-BI), and apolipoprotein (ApoE) are important host receptors for HCV entry and infection^47^. However, antibodies targeting receptor-mediated entry of HCV into hepatocytes confer limited therapeutic benefits^48^, raising the possibility that other mechanisms can mediate virus transmission. Recently, it has been reported that exosomes could mediate CD81-, SR-BI- and ApoE receptor-independent transmission of HCV infection^38^, which could explain in part why neutralization antibodies or therapies targeting host/viral protein interactions at the level of cell entry are compromised and likely occur via cell-to-cell transmission by exosomes. While evidence suggests that exosomes can transfer genetic materials between cells, their role in immune dysregulation, particularly, in MDSC induction remains obscure. Based on these studies, we first tested whether the presence of anti-CD81, anti-SR-BI or anti-ApoE antibodies would block the fusogenic effect of exosomes on myeloid cells. To this end, we incubated CD33^+^ myeloid cells with these antibodies or IgG control for 2 h, respectively, then added PKH67-labeled HCV-Exo into the cultures for 1 h, followed by flow cytometric analysis of exosome association with myeloid cells. In this case, CD33^+^ cells incubated with exosomes without PKH67 labeling or with PKH67 labeling only served as a negative control (NC) and a positive control (PC), respectively. As shown in Supplementary Fig. S1a, we found that anti-CD81, anti-SR-BI and anti-ApoE pretreatment could not block the exosome uptake by myeloid cells. We then assessed whether these blocking antibodies could functionally affect the exosome-induced MDSC induction. To this end, 5 healthy PBMCs were pretreated with these antibodies or IgG control for 2 h, then HCV-Exo were added to the cultures for 3 days, followed by flow cytometric analysis of CD33^+^ myeloid cells. As shown in Supplementary Fig. S1b, while anti-SR-BI did not change the frequency of myeloid cells in PBMCs in the presence of HCV-Exo, anti-CD81 and anti-ApoE pretreatment remarkably reduced the frequency of myeloid cells in PBMCs exposed to HCV-Exo. These results were not in line with the data from the experiment of blocking exosome uptake; we thus questioned whether these decreases in CD33^+^ myeloid cells were due to the effect of the blocking antibodies on PBMCs. To depict the disparity shown in Supplementary Fig. S1a and b, we incubated PBMCs with blocking antibodies in the absence of HCV-Exo and cultured the cells under the same condition. We observed the same decreases in CD33^+^ myeloid cell frequencies in antibody (Ab)-treated PBMCs (Supplementary Fig. S1c), suggesting anti-CD81 and anti-ApoE antibodies could alter the myeloid cell behavior. Taken together, these data indicate that HCV-Exo mediate a CD81-, SR-BI- and ApoE receptor-independent effect on MDSC induction during viral infection.

**Fig. 5:**
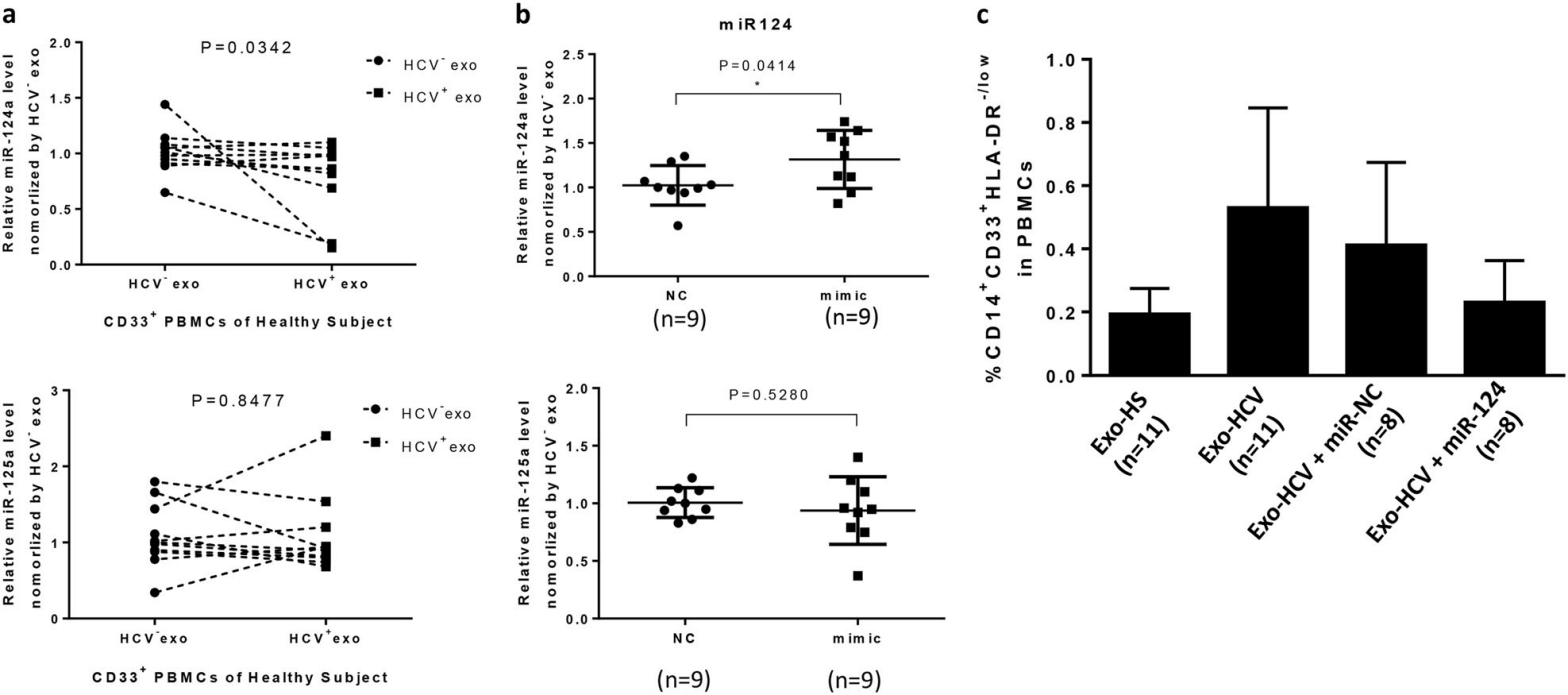
HCV-Exo induce MDSCs through the miR-124-mediated signaling pathway. **a** miR-124 (upper panel) and miR-125 (lower panel) expressions in CD33^+^ myeloid cells treated with exosomes isolated from the supernatants of HCV-infected (Huh7R) and -uninfected (Huh7) hepatocytes. **b** Human PBMCs were treated with exosomes isolated from HS, with or without transfection of miR-124 mimic or miR-NC (negative control) for 3 days, followed by RT-PCR analysis of miR-124 or miR-125 levels. Each symbol represents one HS PBMC to be treated (*n* = 9 in each group). **c** MDSC inductions in healthy PBMCs treated with exosomes isolated from plasma of HS and HCV patients (*n* = 11 in each group) with the centrifugation method, with or without transfection of miR-124 or miR-NC (*n* = 8 in each group), and cultured for 3 days, and MDSCs were analyzed by flow cytometry

**Fig. 6:**
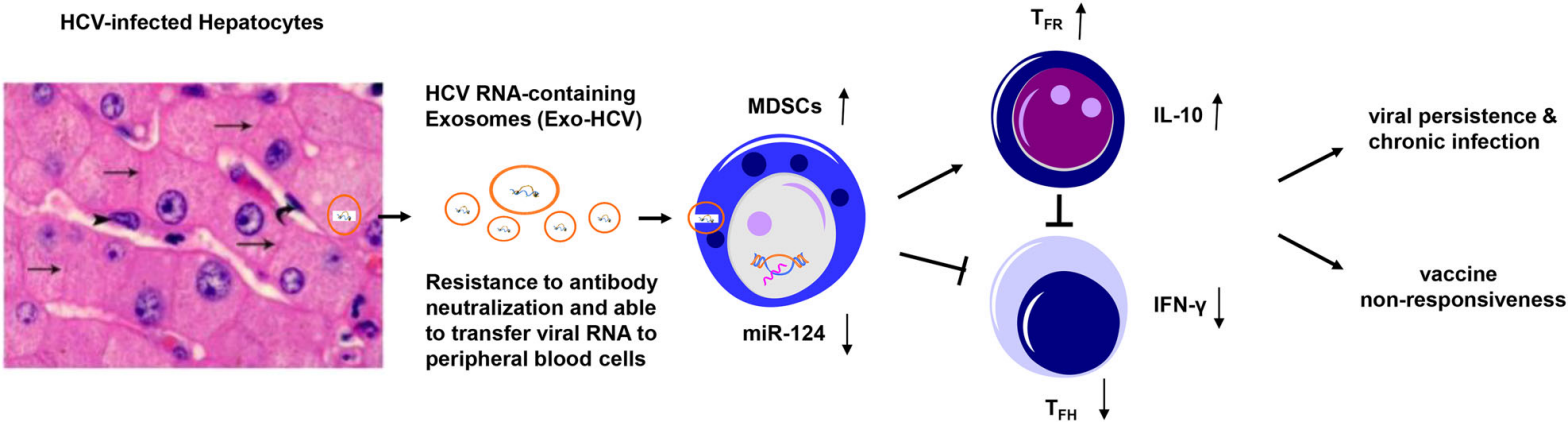
A schematic model for HCV-associated exosomes in promoting MDSC expansion to regulate T follicular cell differentiation and function. HCV-infected hepatocytes can secrete HCV RNA in forms of exosomes, which are more resistant to neutralizing antibodies in the peripheral blood. These HCV RNA-containing exosomes can be taken up by monocytic myeloid cells and promote MDSC expansion via inhibiting miR-124 expression which, in turn, regulates the differentiation of T_FR_ cells along with increasing IL-10 production and suppression of IFN-γ secretion from T_FH_ cells. This paradigm represents a novel mechanism of immune dysregulation by HCV-Exo, leading to viral persistence and vaccine non-responsiveness during chronic viral infection

Using gene array and functional assays, we have previously shown that decline of miR-124 in myeloid cells promotes Treg cell development during HCV infection;^26^ however, how HCV infection leads to miR-124 downregulation in myeloid cells remains unknown. Notably, we did not observe any difference of miR-124 levels in HCV-Exo compared to HS-Exo, suggesting that the observed decline of miR-124 expression in myeloid cells^26^ is not due to its low level in exosomes secreted from HCV-infected cells, but may be due to the effect of HCV-Exo on myeloid cells. To further investigate the mechanisms of HCV-Exo in MDSC induction, we incubated healthy CD33^+^ myeloid cells for 3 days with exosomes purified from the culture media of HCV-infected or non-infected Huh7 cells, followed by real-time RT-PCR to measure miRNA expression profiles. As shown in Fig. 5a, miR-124, but not miR-125, was significantly inhibited in CD33^+^ myeloid cells by HCV RNA-containing exosomes isolated from the supernatant of HCV-infected Huh7R when compared to those exposed to exosomes derived from Huh7 cells without HCV infection. To investigate whether reconstitution of miR-124 in PBMCs can change the ability of HCV-Exo to induce MDSC expansion, we treated human PBMCs transfected with or without miR-124 mimic or negative control for 3–5 days with exosomes isolated from HCV patients or HS, followed by analysis of miR-124/miR-125 levels and MDSC frequencies. As shown in Fig. 5b, compared to the cells transfected with miR-NC, PBMCs transfected with miR-124 mimic exhibited an increase in miR-124, but not miR-125, level in CD33^+^ myeloid cells. PBMCs incubated with HCV-Exo induced monocytic MDSC (CD14^+^CD33^+^HLA-DR^−^) development compared to those treated with HS-Exo. Importantly, reconstituting PBMCs with miR-124 abrogated the effect of HCV-Exo in inducing the expansion of MDSCs (Fig. 5c) and abolished the induction of MDSCs by exosomes derived from the culture supernatants of HCV-infected hepatocytes. In conjunction with our previous studies, these new findings suggest that HCV-infected hepatocytes can secrete HCV RNA in the form of exosomes, and these HCV RNA-containing exosomes can be taken up by monocytic myeloid cells to trigger their differentiation into MDSCs via inhibiting miR-124. These MDSCs, in turn, can promote the expansion of Foxp3^+^ Tregs, including T_FR_ differentiation, and thus regulate IFN-γ and IL-10 production in CD4^+^CXCR5^+^ T follicular cells, which are important for viral persistence and vaccine non-responsiveness during infection, as depicted in Fig. 6.

## Discussion

We have previously shown that MDSCs expand during chronic viral (HCV, HIV) infection and regulate T-cell differentiation and functions^20,25^. We have also demonstrated that a decline in miR-124 expression enhances Foxp3^+^ Treg cell differentiation and inhibits effector T (Teff) cell functions via regulating the signal transducer and activator of transcription 3 (STAT-3) pathway^26^. Our miRNA array analysis and RT-PCR assays show that miR-124 is downregulated in MDSCs during HCV infection. Bioinformatic analysis suggests that miR-124 may be involved in the regulation of STAT-3, which is overexpressed in MDSCs from HCV-infected patients^26^. Notably, reconstitution of miR-124 decreases the levels of STAT-3 as well as IL-10 and transforming growth factor-β, which are also overexpressed in MDCSs and reduces the frequencies of Foxp3^+^ Treg cells that are developed during chronic HCV infection^26^. In this study, we further demonstrate that: (1) chronic HCV infection is associated with a decrease in T_FH_ cells with relatively low IL-21 expression, and remarkably increased T_FR_ cells with elevated IL-10 production; (2) HCV-associated MDSCs can inhibit the function of T_FH_ cells via promoting the induction and/or maintenance of T_FR_ cells during chronic HCV infection; (3) exosomes derived from the plasma of HCV patients as well as HCV-infected hepatocytes contain HCV RNA that can induce MDSC expansion, but exosomes isolated from patients received antiviral treatment with SVR do not harbor HCV RNA and cannot induce MDSC development; (4) HCV-infected hepatocytes can secrete HCV RNA in the form of exosomes, and these HCV RNA-containing exosomes can be taken up by monocytic myeloid cells in a virus receptor-independent way and regulate their differentiation into M-MDSCs via inhibiting miR-124. These MDSCs then promote the expansion of Foxp3^+^ Tregs, including T_FH_ differentiation, and thus inhibit T_FH_ cell functions, including IFN-γ and IL-10 secretion. These results reveal a novel paradigm regarding the role of HCV-Exo in promoting the expansion of MDSCs and Treg (T_FR_) cells as a mechanism of inhibiting Teff (T_FH_) cell functions during chronic HCV infection.

Exosomes are enriched in tetraspanins and have fusogenic capabilities^27,28^. They are formed by membrane budding into the lumen of an endocytic compartment, leading to the formation of multivesicular bodies (MVBs). Fusion of MVBs to the plasma membrane leads to the extracellular release of exosomes, a process similar to an enveloped virus particle budding from the host cell surface. Indeed, the budding of several enveloped viruses (HCV, HBV and HIV) involves an endosomal sorting complex required for transport (ESCRT), which is part of the cellular machinery used to form MVBs^49,50^. Since human tetraspanin CD81 binds the HCV envelope glycoprotein E2, exosomes may play an important role in the cellular and extracellular trafficking of HCV particles. In line with this, previous studies of the HCV life cycle revealed that in the absence of CD81, HCV envelope proteins are almost completely retained in the endoplasmic reticulum. When CD81 is present, a fraction of HCV envelope proteins passes through the Golgi apparatus, matures as it acquires complex sugars and is extracellularly associated with exosomes^36^. It has also been reported that Hrs, an ESCRT-0 component, is critical for assembling and budding of HCV through the exosomal secretion pathway^50^. However, as other investigators have failed to detect HCV proteins in exosomes, we also could not detect HCV E1, E2 and Core proteins in exosomes isolated from plasma of HCV-infected patients and supernatant of HCV-infected hepatocytes, using either ultracentrifugation isolation method or purification by immune capture of exosomes with beads coated with anti-CD81 antibodies. This may be due to the small amount of HCV mature/immature particles in exosomes purified from the plasma or supernatant, and/or the limited sensitivity of our detection methods. Nevertheless, we found HCV RNA present in exosomes from both the HCV patient plasma and the supernatants of HCV-infected hepatocytes, but not in exosomes isolated from HCV patients who received DAA treatment with SVR. Importantly, exosomes are equipped to survive extracellularly to perform their functions, as suggested by the finding that they contain CD55 and CD59 to protect cell membrane-embedded materials from complement attack^51^. We thus propose that “bona fide” HCV particles replicate and exit the infected hepatocytes in the form of exosomes, which then circulate and exploit their fusogenic capabilities to other cells, even in the presence of complement and neutralizing antibodies. This may explain, at least in part, the relative ineffectiveness of neutralizing antibodies in blocking HCV infection, as exosomes containing HCV RNA may fuse to uninfected cells or replication-non-permissive bystander immune cells in the presence of virus-specific antibodies.

While the exact physiological functions of exosomes remain unclear, they have been implicated in transferring materials (e.g., antigens, cytokines and chemokines, mRNAs or miRNAs) among cells without direct cell-to-cell contact^29–31^. Exosomes can fuse to various cell types, given their content of several membrane proteins, such as α4β1 integrins, ICAMs (intercellular adhesion molecules), tetraspanins and MHC (major histocompatibility complex)^52^. The potential physiological relevance of our finding of HCV RNA transfer via exosomes is supported by recent reports showing that exosome-mediated cell–cell transmission of HCV RNA, miRNA and small interfering RNA occurs between hepatocytes in vivo^38,53^, that HCV RNA-containing exosomes are detected in the plasma of chronically HCV-infected patients^36^ and that pDCs are abundant in the HCV-infected liver and respond normally to HCV-infected cells^54,55^.

The current concept of virus-induced innate immune responses is that recognition of viral nucleic acids typically occurs within cells that are either productively infected or have internalized viral particles. For example, HCV has evolved a potent strategy that precludes type I IFN induction by infected hepatocytes^56,57^. Recently, it has been reported that short-range exosomes can transfer HCV RNA from infected cells to uninfected hepatocytes or pDCs and trigger innate immunity^37–39^. The exosomal viral RNA transfer is independent of CD81-, SR-BI- and ApoE receptor-mediated mechanisms, but depends on the ESCRT machinery and on Annexin A2, an RNA-binding protein involved in membrane vesicle trafficking, and is suppressed by exosome release inhibitors. Additionally, purified concentrated HCV RNA-containing exosomes were sufficient to transfer HCV infection in hepatocytes and to activate interferon responses in pDC immune cells^37–39^. In this study, we demonstrated that HCV-Exo can be taken up by monocytic myeloid cells in a virus receptor-independent manner to inhibit miR-124 expression in monocytic myeloid cells and promote their differentiation into MDSCs, which in turn, regulate T_FR_ and T_FH_ differentiation and functions. As depicted in Fig. 6, our current results illustrate a previously unrecognized mechanism of MDSC expansion and T_FR_ regulation, in which the vesicular transport machinery in HCV-infected cells produces viral RNA-loaded, immunomodulatory exosomes. This may serve as a viral strategy to evade pathogen sensing, as well as a host strategy to induce an unopposed innate-to-adaptive immune response in replication-non-permissive bystander cells.

Our findings have potential implications in relation to HCV-induced immune dysregulation and, most importantly, vaccine responses. For example, we and others have previously reported poor vaccine responses in virally infected individuals^58–61^. We have also shown that chronically HCV-infected individuals exhibit premature T-cell aging, contributing to the poor HBV vaccine response in HCV- and/or HIV-infected individuals^62,63^. It is well known that cellular and humoral immunity wanes with age^64^, although the exact mechanisms for this process remain unclear. A recent report, however, revealed that defective T cell-dependent B-cell responses associated with aging can result from an overabundance of fully suppressive T_FR_ cells and impaired T_FH_ cell functions^65^. Notably, inhibitory T_FR_ cells expand with age, leading to higher T_FR_/T_FH_ ratios, and these impaired T_FH_ and increased T_FR_ cells lead to diminished B-cell responses in the elderly. In this study, we further demonstrate that HCV-Exo-induced MDSCs contribute to an altered T_FR_/T_FH_ cell ratio and IL-10/IFN-γ production, suggesting that chronic viral infection-caused premature immune aging recapitulates the nature aging process in the elderly. This may underpin, at least in part, the mechanism of vaccine non-responsiveness in virally infected individuals.

In summary, we have shown that HCV-associated exosomes contain immunomodulatory viral RNA and serve as vehicles to transmit HCV RNA from infected cells to bystander immune cells and are sufficient to induce MDSC expansion via inhibiting miR-124 to regulate T follicular cell differentiation and functions. Our results illustrate an unopposed mechanism of viral immune evasion: i.e., exosomal package and secretion of viral RNA from infected cells may represent a perfect pathogen–host interaction and immune evasion machinery that serves as a viral strategy to transmit infection in a cell contact-independent manner as well as a host strategy to induce innate-to-adaptive immunity by a viral replication-non-permissive bystander mechanism. Further characterization of the virus-associated exosomes in regulation of the host immunity will enhance our understanding of the mechanisms of virus persistence and vaccine failure in chronic viral infection in humans. Delineating these mechanisms have clinical implications in designing prophylactic/therapeutic vaccines to combat HCV and other chronic viral infections, and may also have broad impact on immunocompromised hosts, such as those with immunosuppressive medications, hemodialysis, organ transplantation and cancer.

## Materials and methods

### Subjects

The study protocol was approved by an institutional review board at East Tennessee State University and James H. Quillen VA Medical Center (ETSU/VA IRB, Johnson City, TN). The study subjects were composed of three populations: 98 chronically HCV-infected patients (70% genotype 1, 30% genotype 2 or 3, viral load range from 21,000 to 48,000,000 IU/ml), 10 HCV-resolved patients after DAA treatment with SVR and 72 age-matched HS. Written informed consent was obtained from all participants. HCV-infected patients were HCV RNA positive, prior to antiviral treatment. Age-matched HS, derived from Physicians Plasma Alliance (PPA), Gray, TN, were negative for HBV, HCV and HIV infection.

### Cell isolation, culture and flow cytometric analysis

PBMCs were isolated from whole blood by Ficoll density gradients (GE Heathcare, Piscataway, NJ). CD33^+^ myeloid cells were further isolated or depleted, depending on the experimental requirements, from PBMCs using the CD33^+^ Cell Isolation Kit and a MidiMACS™ Separator (Miltenyi Biotec Inc., Auburn, CA). The isolated cells were cultured in RPMI 1640 medium containing 10% fetal bovine serum (FBS; Atlanta Biologicals, Flowery Branch, GA), 100 IU/ml penicillin and 2 mM l-glutamine (Thermo Scientific, Logan, Utah) at 37 °C and 5% CO_2_ atmosphere. Co-culture of cells with or without exosomes was carried out as described in the Results. Phenotypic and intracellular cytokine analyses of PBMCs or co-cultured cells, with or without stimulation, were carried out as previously reported^25,26^, using the following reagents: anti-CD4-FITC, anti-CXCR5-AF 647, anti-Foxp3-PE-Cy5, anti-IL-21-PE, anti-IL-10-PE, anti-IFN-γ-PE, anti-CD33-APC, anti-CD14- PerCP-Cy5.5, anti-HLA-DR-FITC and anti-CD81-PE, or isotype control antibodies (BD Bioscience, San Jose, CA). To see if exosomes can be taken up by myeloid cells, CD33^+^ cells were incubated with the CFSE-labeled HCV-Exo for 1 h; after intensive washing, flow cytometry was employed to examine the fluorescence signal within the cells. For exosome uptake blocking assays, the purified CD33^+^ myeloid cells were incubated with blocking antibodies (mouse anti-CD81, sc-23962, Santa Cruz; rabbit anti-SR-BI, ab-525629, Abcam; Goat anti-ApoE, AB947, EMD Milipore) and isotype control (mouse IgG 553447, BD; rabbit IgG sc-2027, Santa Cruz; goat IgG 31245, ThermoFisher) for 2 h, respectively; PKH67 (MINI67, Sigma)-labeled HCV-Exo were added into the culture for 1 h. Cells cultured with HCV-Exo without labeling or with PKH67-labeled HCV-Exo only serve as NC or PC. To functionally analyze the effect of blocking antibodies on HCV-Exo-mediated myeloid cell differentiation, PBMCs were incubated with the blocking antibodies and control IgG for 2 h, respectively, and cultured in the presence or absence of HCV-Exo for 3 days, followed by flow cytometric analysis for the frequency of CD33^+^ myeloid cells in PBMCs. The cells were acquired on an AccuriTM C6 flow cytometer (BD, Franklin Lakes, NJ) and analyzed using FlowJo software (Tree Star, Inc., Ashland, OR). The fluorescence minus one (FMO) strategy was used to determine background levels and to adjust multicolor compensation for cell gating.

### Exosome isolation and purification

Plasma was isolated from 50 ml of blood from the research subjects and filtered to exclude particles larger than 0.8 μm using sytinge filters (Millipore® Millex®-AA Cat. No: SLAA033SS, Billerica, MA). Supernatants of the culture media from Huh7 cells with or without HCV infection were also filtered in the same way. Exosomes were isolated from 4 ml of filtered plasma or supernatant with exoEasy Maxi Kit (QIAGEN Cat. No. 76064) per the manufacturer’s instructions. To further purify exosomes and exclude other microparticles or free HCV contamination, pre-enriched exosomes were incubated with Mix CD81 beads (Invitrogen Cat. No: 00353003) at 4 °C overnight, and then the bead-bound exosomes were purified with magnetic separator according to the protocol of the manufacturer. Additionally, exosomes were also isolated by the differential centrifugation method as previously reported^36^. In brief, plasma from HCV^+/−^ subjects or culture media from HCV^+/−^ Huh7 cells were centrifuged at 200 × *g* for 10 min (P1), the supernatants were collected and centrifuged twice at 500 × *g* for 10 min (P2), the second supernatants were collected and centrifuged twice at 2000 × *g* for 15 min (P3), the third supernatants were collected and centrifuged once at 10,000 × *g* for 30 min (P4), the fourth supernatants were collected and centrifuged once at 70,000 × *g* for 60 min (P5), and the precipitates (exosomes) were resolved in 300 μl distilled water for further experiments.

### Western blot

Exosomes purified by CD81-bound beads were lysed with RIPA buffer with sonication. Western blot was carried out as described previously using anti-CD63 (Santa Cruz Biotechnology Cat. No: sc-5275, Santa Cruz, CA), anti-CD81 (Santa Cruz Cat. No: sc-9185, Santa Cruz, CA) and anti-HCV core (ThermoFisher, C7-50, Waltham, MA) primary antibodies, followed by a horseradish peroxidase-conjugated secondary antibody (Cell Signaling)^25,26^. Membranes were stripped and re-probed with anti-GAPDH antibody (Santa Cruz Biotechnology) as a loading control. The proteins were visualized using the Amersham ECL Prime Western Blotting Detection Reagents (GE Healthcare Biosciences, Pittsburgh, PA) and Bio-Rad chemiDoc^MP^ imaging system (Philadelphia, PA).

### Confocal microscopy and IEM

CD33^+^ myeloid cells were isolated and cultured with CFSE-labeled HCV-Exo for 1 h, followed by examination of fluorescence signal with confocal laser-scanning inverted microscope (Leica Confocal Model TCS sp8, Germany). To observe the structure of exosomes, the purified exosomes (P5 preparation) were seeded on carbon-coated Formvar nickel grids (EM Sciences, Hatfield, PA) and left to adsorb onto grids at 4 °C overnight, fixed with EM-grade 2% paraformaldehyde (EM Sciences, Hatfield, PA) and 0.1% glutaraldehyde (EM Sciences, Hatfield, PA) at 4 °C for 1 h, blocked with 2% cold fish gelatin (Sigma, St. Louis, MO)/0.1% bovine serum albumin/phosphate-buffered saline (PBS) for 30 min at room temperature and incubated with 50 µg/ml mouse anti-human CD81 antibody (BD Pharmingen, San Jose, CA) for 2 h at room temperature. After washing with PBS, the exosomes were incubated with 1:40 diluted 12 nm Colloidal Gold-AffiniPure Goat anti-mouse IgG (Jackson ImmunoResearch Laboratories, West Grove, PA) for 30 min at room temperature. After washing with PBS, the grids were stained with 1% aqueous uranyl acetate before observation with a Zeiss Auriga 40 operating in STEM mode at 30 kV electron microscope.

### Real-time PCR

RNA was isolated from purified exosomes or CD33^+^ cells incubated with exosomes using Total Exosome RNA and Proteain Isolation Kit (Cat. No. 4478545, Invitrogen). RNA quality and quantity were analyzed using a BioPhotometer spectrophotometer ultraviolet–visible. Reverse transcription was performed using High-Capacity Complementary DNA Reverse Transcription Kits (Applied Biosystems, Foster City, CA). An HCV Genesig^®^ Standard Kit was employed to perform real-time PCR measuring 5’ untranslated region of HCV genome. HCV RNA copy numbers were calculated based on the plasma or supernatant volume to be used for isolation of exosomes and the standard curve of the positive control according to instructions of the manufacturer (Primerdesign^TM^ Ltd, Chandler’s Ford SO53 4DG, UK). Quantifications of miR-124, miR-125 and miR-29 levels were completed using Power SYBR® Master Mix Kit (Cat. No. 4367659, Applied Biosystem) on CFX96TM Real-time PCR Detection System (Bio-Rad Laboratories, Inc. Hercules, CA) according to the protocols of the manufacturers. After agarose gel electrophoresis, image was captured using Bio-Rad chemiDoc^MP^ imaging system (Philadelphia, PA). miRNA levels were quantified with the 2^−ΔΔct^ relative quantification method, normalized to U61 small nuclear RNA (SNORD61).

### miRNA transfection

PBMCs of HCV patients were transfected with 30 pmol of mirVana microRNA mimic for miR-124 or negative control #1 (Life technologies, Grand Island, NY) using the Human Monocyte Nucleofector Kit and Nucleofector I Device (Lonza, Allendale, NJ). We could achieve 60% transfection efficiency with fluorescence-labeled miRNAs with this method^26^. The transfected cells were cultured in 10% FBS Iscove's modified Dulbecco's medium in the presence of exosomes isolated from plasma of HCV patients or HS and from supernatants of HCV-infected or -uninfected hepatocytes (Lonza, Allendale, NJ) for 3 and 5 days, followed by immune staining for the frequency of MDSCs as described above.

### Statistical analysis

The parametric data were presented as mean ± SEM. Association between MDSCs and T_FR_ or T_FH_ was analyzed based on their frequencies in peripheral blood by Pearson's correlation. Comparisons between groups were made by one-way analysis of variance using Prism version 5. One-tail paired *t*-test was used to compare two groups with transfection by miR-124 mimic and negative control. The nonparametric data were presented as median and analyzed by one-tail Mann–Whitney test. *P* < 0.05 or *P* < 0.01 were considered significant or very significant, respectively.

## Electronic supplementary material

Supplementary Information

## Electronic supplementary material

**Supplementary Information** accompanies the paper at (10.1038/s41421-018-0052-z).
