# Supplementary material for: HCV-associated exosomes promote myeloid-derived suppressor cell expansion via inhibiting miR-124 to regulate T follicular cell differentiation and function
Source: Cell Discov. 2018 Sep 11;4:51. doi: 10.1038/s41421-018-0052-z (PMC6131392; doi:10.1038/s41421-018-0052-z)
Supplement: Supplementary file 1 — Supplementary Information [file 41421_2018_52_MOESM1_ESM.pdf]

Supplementary Fig. S1

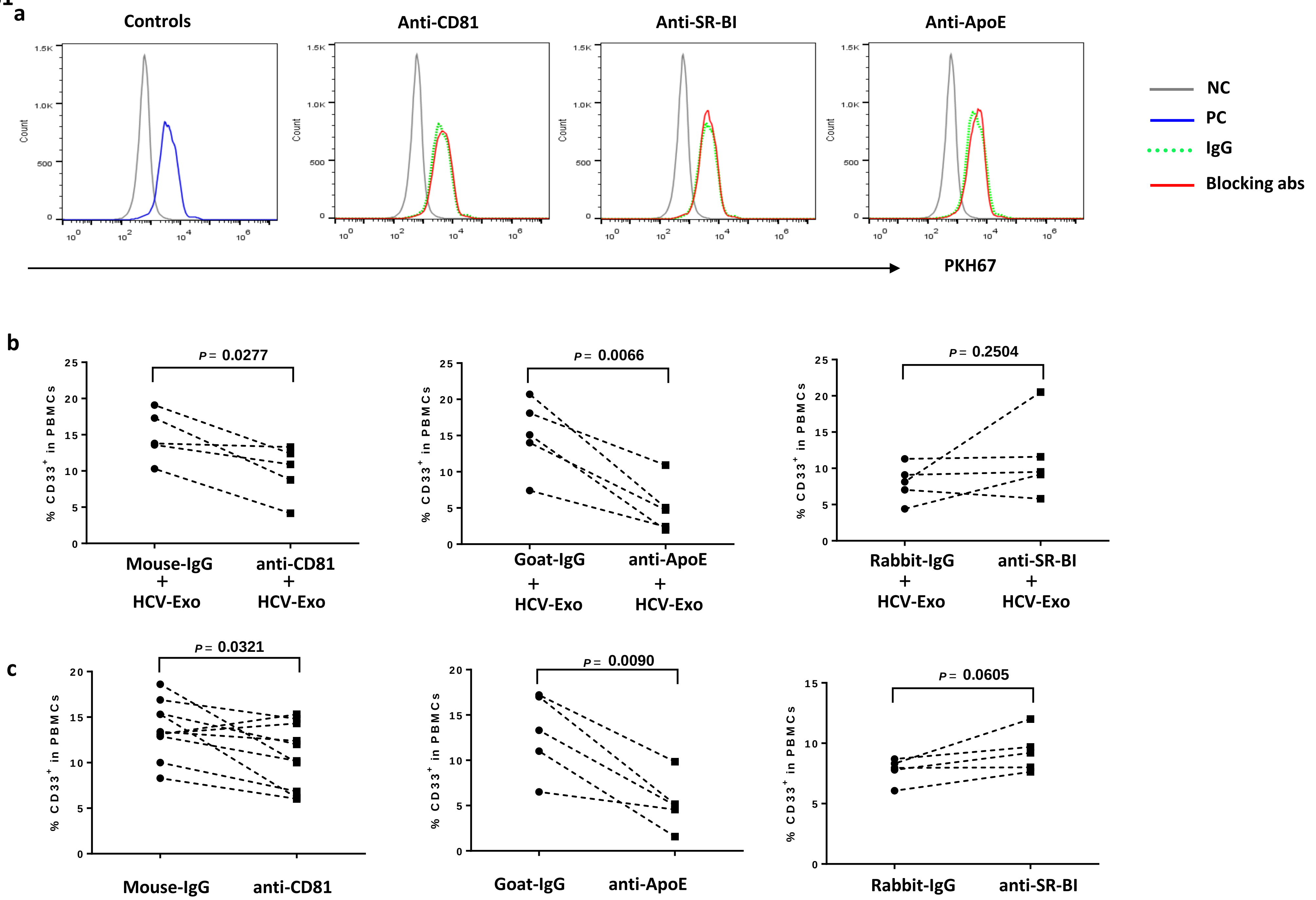

Supplementary Fig. S1 The role of CD81, SR-BI, and ApoE receptors in HCV-Exo entry and effect on myeloid cells. a Blocking HCV-Exo uptake by myeloid cells. Purified $CD33^+$ cells were incubated with anti-CD81, SR-BI, and ApoE blocking antibodies or isotype control IgG for 2 h, respectively, and then the PKH67-labelled HCV-Exo were added into the culture for 1 h, after intensive washing, the cells was analyzed by flow cytometry for fluorescence signal. Cells incubated with unlabeled HCV-Exo or PKH67-labelled HCV-Exo alone without blocking antibodies serve as negative control (NC) and positive control (PC). Representative overlaid histograms of the flow data are shown. **b, c** Functional blocking the effect of HCV-Exo on myeloid cell differentiation. PBMCs were incubated with anti-CD81, SR-BI, and ApoE blocking antibodies or isotype control IgG for 2 h, respectively, and cultured in the presence (B) or absence (C) of HCV-Exo for 3 days. The frequency of $CD33^+$ myeloid cells in PBMCs were analyzed by flow cytometry.
